# Targeted gene knockdown validates the essential role of lactate dehydrogenase in *Cryptosporidium parvum*

**DOI:** 10.1016/j.ijpara.2017.05.002

**Published:** 2017-11

**Authors:** William H. Witola, Xuejin Zhang, Chi Yong Kim

**Affiliations:** Department of Pathobiology, College of Veterinary Medicine, University of Illinois, Urbana-Champaign, USA

**Keywords:** *Cryptosporidium parvum*, Phosphorodiamidate morpholino oligomers, Gene knockdown, Lactate dehydrogenase function

## Abstract

*Cryptosporidium parvum* is a zoonotic protozoan that can cause a life-threatening gastrointestinal syndrome in children and in immunocompromised adults. Currently, the only approved drug for treatment of *Cryptosporidium* infections in humans is nitazoxanide, but it is not effective in immunocompromised individuals or in children with malnutrition. This is compounded by the lack of genetic methods for studying and validating potential drug targets in the parasite. Therefore, in this study, we endeavoured to adapt the use of a phosphorodiamidate morpholino oligomer (morpholino) antisense approach to develop a targeted gene knockdown assay for use in *C. parvum*. We show that morpholinos, at non-toxic concentrations, are rapidly internalised by both *C. parvum* and host cells (HCT-8), and distribute diffusely throughout the cytosol. Using morpholinos to separately target *C. parvum* lactate dehydrogenase and putative arginine n-methyltransferase genes, within 36 h of in vitro culture, we achieved over 10-fold down-regulation of the respective encoded proteins in *C. parvum*. Pursuant to this, we observed that knockdown of *C. parvum* lactate dehydrogenase produced a dramatic reduction in intracellular growth and development of *C. parvum* by 56 h of culture. On the other hand, *C. parvum* putative arginine n-methyltransferase knockdown did not appear to have any effect on parasite growth, but nevertheless provided the proof-of-principle that the morpholino knockdown assay in *C. parvum* was consistent. Together, our findings present a gene regulation approach for interrogating gene function in *C. parvum* in vitro, and further provide genetic evidence for the essential role of *C. parvum* lactate dehydrogenase in fueling the growth and development of intracellular *C. parvum*.

## Highlights

• Morpholino oligomers antisense approach was developed as a reverse genetic tool in *Cryptosporidium parvum*.
• Significant knockdown of *C. parvum* LDH and arginine methyltransferase was achieved using gene-target morpholinos.
• Knockdown of *C. parvum* LDH dramatically decreased growth of *C. parvum* in vitro.

## Graphical abstract

**Figure:**
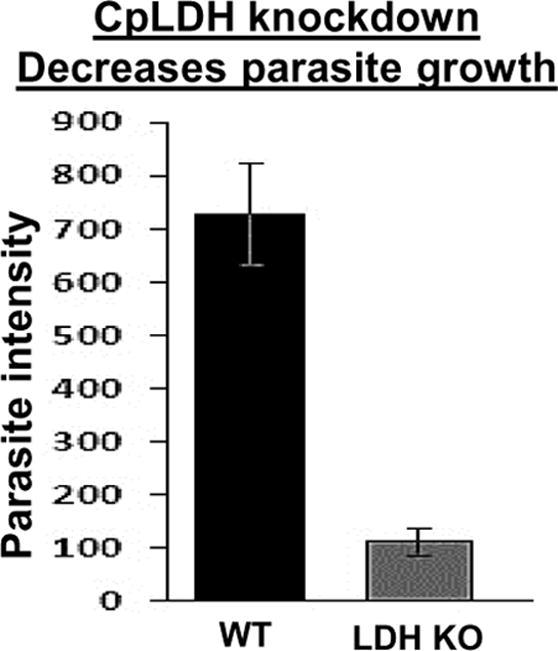

## 1 Introduction

*Cryptosporidium parvum* is a highly prevalent zoonotic and anthroponotic apicomplexan protozoan of medical and veterinary significance. It causes a serious diarrheal syndrome (cryptosporidiosis) in calves, lambs and goat kids, resulting in poor growth rates and high neonatal mortality (De Graaf et al., 1999, Jex and Gasser, 2009, Karanis et al., 2010). In humans, *Cryptosporidium* spp. (*C. parvum* together with *Cryptosporidium hominis*) are the number two major cause of diarrheal disease in young children resulting in significant childhood morbidity and mortality in developing countries (Checkley et al., 2015). The parasite is mostly transmitted through contaminated water sources and by contact with infected animals. A compounding problem of *Cryptosporidium* infection is the unavailability of fully effective drugs or vaccines against it (De Graaf et al., 1999, Benitez et al., 2009, Cabada and White, 2010, Bouzid et al., 2013). Moreover, *Cryptosporidium* oocysts contaminating the environment are difficult to eliminate because they are resistant to most chemical disinfectants, as well as to commonly used water treatments such as chlorination (Macarisin et al., 2010).

Although *C. parvum* is widespread worldwide, little is known about the biology of this parasite at genetic and molecular levels due to the extremely limited genetic tools for studying it (Bouzid et al, 2013)*.* Nevertheless, recently a clustered regularly interspaced short palindromic repeat (CRISPR)/Cas9 gene knockout system for *Cryptosporidium* was reported (Vinayak et al., 2015). Indeed, development of safe and effective drugs against *Cryptosporidium* will require identification and validation of molecular targets using genetic tools (Checkley et al, 2015). Thus, in the present study, we endeavoured to adapt the use of a phosphorodiamidate morpholino oligomers (morpholinos) antisense approach to develop a targeted gene knockdown assay to study and validate gene function in *C. parvum*. Morpholinos are synthetic chains of six-membered non-ionic DNA analogues, with neutral and hydrophilic phosphorodiamidate groups that enhance stability and solubility (Moulton et al., 2004). Morpholinos are attached at the 3′-end to a membrane-penetrating octa-guanidine dendrimer that allows them to readily cross multiple membrane barriers (Li and Morcos, 2008). They block RNA splicing or initiation of protein translation by Watson/Crick base-pairing with target RNA (Morcos, 2007), and have been successfully adapted for gene silencing in various organisms including protozoa and nematodes (Lai et al., 2012, Witola et al., 2014, Witola et al., 2016, Garg et al., 2015). The resilience of morpholinos provides an opportunity for developing gene regulation approaches for the in vitro culture stages as wells as the in vivo life stages of *Cryptosporidium*. Herein, we report the development of an in vitro assay for targeted gene knockdown in *C. parvum* using morpholinos. Using this assay, we targeted the knockdown of the *C. parvum* lactate dehydrogenase gene (CpLDH) and provide genetic evidence that it plays an important role during the intracellular growth of *C. parvum* in vitro.

## 2 Materials and methods

### 2.1 *Cryptosporidium parvum* cDNA synthesis

Freshly extracted and purified *C. parvum* (AUCP-1 isolate) oocysts suspended in PBS were generously provided by Dr. Mark Kuhlenschmidt of the University of Illinois at Urbana-Champaign, USA. Approximately 10^5^ of the oocysts were pelleted by centrifugation and total RNA extracted using the Trizol reagent (Life Technologies, USA) following the manufacturer’s protocol. Approximately 1 μg of the total RNA was treated with DNase I (Invitrogen, USA) to remove residual genomic DNA, followed by reverse transcription using the iScript Select cDNA Synthesis kit (Bio-Rad, USA) according to the manufacturer’s instructions.

### 2.2 Cloning of CpLDH and CpAMT coding sequences

The primer pair for amplification of CpLDH coding sequence (GenBank accession number AF274310.1) was 5′-*CTCGAG***ATG**ATTGAAAGACGCAAGA-3′ (Forward, with the *Xho*I restriction site italicised and start codon in bold) and 5′-*GGATCC***TTA**TGCTCCAGCTGGT-3′ (Reverse, with the *Bam*HI site italicised and stop codon in bold). The primer pair for the *C. parvum* putative arginine n-methyltransferase (CpAMT) coding sequence (CryptoDB genome database identification number: Cgd8_4760) was 5′-*CTCGAG***ATG**ATTAAACCAACAGAAGAAGAGCTT-3′ (Forward, with the *Xho*I restriction site italicised and start codon in bold) and 5′-*GGATCC***TTA**CCTTAGTCTATAATAGTTAATCCT-3′ (Reverse, with the *Bam*HI site italicised and stop codon in bold). The *Xho*I and *Bam*HI restriction sequences were introduced in the forward and reverse primers, respectively, for site-directed cloning at the *Xho*I/*Bam*HI site of the pET15b expression vector (Novagen, USA). The coding fragments were generated by PCR from *C. parvum* cDNA using high fidelity DNA polymerase (Affymetrix, USA) and cloned into the pGEMT vector (Promega, USA) for sequencing to confirm identity. The coding fragments were transferred from the pGEMT vector by dual excision with *Xho*I and *Bam*HI restriction enzymes followed by cloning at the *Xho*I*/Bam*HI site of the pET15b expression vector in-frame with the hexahistidine (His-tag) at the N-terminal. The recombinant expression vectors were amplified in DH5α *Escherichia coli* and transformed into protein expression *E. coli* BL21-CodonPlus-DE3-RIL (Stratagene, USA).

### 2.3 Expression and purification of recombinant CpLDH and CpAMT

Transformed expression *E. coli* for CpLDH or CpAMT was cultured at 37 °C in Luria broth medium (supplemented with 100 μg/ml of ampicillin and 34 μg/ml of chloramphenicol) to an *A*_600_ of 0.8 followed by addition of 1 mM isopropyl-β-d-thiogalactopyranoside to induce protein expression. The expression *E. coli* was harvested by centrifugation and lysed under native conditions by sonicating in lysis buffer (50 mM NaH_2_PO_4_, 300 mM NaCl, 10 mM Imidazole, pH 8.0) containing a 1× EDTA-free protease inhibitor cocktail, 600 units of benzonase and 30 kU of lysozyme (EMD Millipore, USA). The lysate was clarified by centrifugation and the His-tagged recombinant protein purified under native conditions by nickel-affinity chromatography according to the manufacturer's instructions (Novagen). The wash buffer used contained 50 mM NaH_2_PO_4_, 300 mM NaCl and 20 mM Imidazole, pH 8.0, while the elution buffer was composed of 50 mM NaH_2_PO_4_, 300 mM NaCl, 250 mM Imidazole, pH 8.0. The eluates were dialysed using a buffer containing 5 mM Hepes–KOH (pH 7.8) and 0.5 mM DTT. The purities of the recombinant proteins were determined by SDS/PAGE, and the concentration measured using a Qubit 3.0 fluorometer (Life Technologies).

### 2.4 Production of CpLDH and CpAMT antisera

To raise polyclonal antibodies against CpLDH and CpAMT, the purified recombinant proteins were emulsified with FCA (Sigma-Aldrich, USA) and injected into rats at 40 µg of protein per rat. Two subsequent booster immunizations were administered at 2 week intervals with 20 µg of the recombinant proteins emulsified in Freund’s Incomplete Adjuvant. The rats were sacrificed, blood collected by cardiac puncture and sera isolated from the blood. Pre-immune and immune sera were used in western blotting assays to assess the specificity of the antisera against the recombinant proteins. The care and use of rats for experimental procedures in this study was performed in accordance with the guidelines of protocol number 17024 approved by the University of Illinois at Urbana-Champaign, USA.

### 2.5 Purification of CpLDH and CpAMT anti-sera to monospecificity

Purification of the CpLDH and CpAMT anti-sera was done as previously described (Witola et al., 2006). Briefly, 8 ml of slurry of Affi-Gel 15 (Bio-Rad) was washed twice with 50 ml of distilled water followed by two washes in 50 mM NaHCO_3_ buffer. The washed resin was mixed with 4 ml of solution containing 4 mg of purified recombinant protein and incubated at 4 °C for 2 h with shaking. The mixture was centrifuged, the supernatant removed, and the resin washed with PBS followed by addition of 800 µl of glycine (1 M, pH 12) and incubation at 4 °C for 1 h. The mixture was washed twice in 50 ml of PBS, 4 ml of crude anti-serum added, and incubated at 4 °C with shaking overnight. The mixture was packed into a 15 ml column and after washing four times with PBS, the monospecific antibodies were eluted with 15 ml of 0.1 M glycine (pH 12.0). The eluate was collected in 1 ml fractions and neutralised with 150 μl of 1 M Tris, pH 6.8. Immunoblots were performed using the purified antibodies with the affinity-purified recombinant proteins and *C. parvum* protein lysates to ascertain antibody specificity.

### 2.6 Design of peptide-conjugated morpholinos

Morpholinos specific for inhibition of the translation of CpLDH and CpAMT mRNA were designed following the strategy we have used previously (Lai et al., 2012, Witola et al., 2014, Witola et al., 2016). Briefly, each morpholino sequence was designed as a 25-base complement sequence of target mRNA that would specifically bind to its complementary 25-base target site via Watson-Crick pairing and sterically block the translation initiation complex. The mRNA sequences for CpLDH and CpAMT were derived from the CryptoDB database using gene identification numbers Cgd7_480-t26_1 and Cg8_4760, respectively. The morpholino targeting CpLDH mRNA was 5′-CGGCAATCTTGCGTCTTTCAATCAT-′3 (with the start codon underlined), while the CpAMT-target morpholino sequence was 5′-CTCTTCTTCTGTTGGTTTAATCATT-3′ (with the start codon underlined). An off-target standard control morpholino sequence was 5′-CCTCTTACCTCAGTTACAATTTAT-3′. The morpholinos were synthesised with an octa-guanidine dendrimer delivery moiety covalently linked at the 3′-end and named vivo morpholinos, or with a fluorescein tag conjugated at the 3′-end as fluorescent morpholinos (Gene Tools, USA).

### 2.7 Vivo morpholino toxicity assay

To assess the toxic concentrations of vivo morpholinos in HCT-8 cells (that were used as host cells for *C. parvum*), a colorimetric assay using the cell proliferation reagent WST-1 (Roche, USA) for the quantification of cell viability was performed. HCT-8 cells were obtained from the American Type Culture Collection (ATCC) (Item number: CCL244) and cultured in 96-well plates in RPMI 1640 medium without phenol red (Life Technologies), but supplemented with 2 g of sodium bicarbonate per litre, 2.5 g of glucose per litre, 10% FBS (Gibco, USA), 1× antibiotic–antimycotic (Gibco), and 1× sodium pyruvate (Gibco). When the cells were confluent, the old medium was replaced with fresh medium with or without CpLDH, CpAMT or off-target vivo morpholino at concentrations of 0 μM, 5 μM, 7.5 μM, 10 μM, 12.5 μM, 15 μM, 17.5 μM, 20 μM, 25 μM and 30 μM. After 36 h of culture, 20 μl of the cell proliferation reagent WST-1, (for quantification of cell viability) was added to each well, mixed and the plates incubated for 1 h at 37 °C with 5% CO2 in the dark. Following incubation, 150 μL of the medium from each well was transferred to a new 96-well plate and quantification of the formazan dye produced by metabolically active cells was read as absorbance at a wavelength of 420 nm using a scanning multi-well spectrophotometer (Spectra Max 250; Molecular Devices, USA). Three independent assays were performed and the dose–response curves of the means of triplicate assays were generated using GraphPad PRISM software to derive the half maximal inhibitory concentration (IC_50_) of morpholino in HCT-8 cells.

### 2.8 Excystation of *C. parvum* oocysts and purification of sporozoites

Sporozoites were excysted from *C. parvum* oocysts following the method described by Kuhlenschmidt et al. (2016). Briefly, to approximately 1 × 10^8^ purified *C. parvum* oocysts suspended in 500 µl of PBS, an equal volume of 40% commercial laundry bleach was added and then the oocysts in solution incubated for 10 min at 4 °C. The oocysts were washed four times in PBS containing 1% (w/v) BSA and resuspended in Hanks balanced salt solution (HBSS), incubated for 60 min at 37 °C, and mixed with an equal volume of warm 1.5% sodium taurocholate in HBSS. Following excystation for 60 min at 37 °C, the sporozoites, together with the oocyst shells, were collected by centrifugation and resuspended in supplemented RPMI 1640 medium. The sporozoites were purified by passing the suspension through a sterile 5 µM syringe filter (Millex, USA) and enumerated with a hemocytometer.

### 2.9 Analysis of uptake of fluorescent morpholinos by HCT-8 cells and sporozoites

To freshly confluent HCT-8 cells cultured in 96-well plate, 200 µl fresh supplemented RPMI medium was added. To triplicate wells, 4 µl of fluorescein-tagged off-target standard control morpholino solution (final concentration of 10 µM), together with 1.2 µl of Endo-Porter solution, a reagent that facilities delivery of morpholinos into cells (Gene Tools Inc.), were added. To triplicate control wells, 4 µl of PBS and 1.2 µl of Endo-Porter solution were added. After mixing, the cells were incubated for 1 h at 37 °C with 5% CO2 in the dark. The cells were washed four times with 200 µl of PBS and analysed by fluorescence microscopy using differential interference contrast microscopy (DIC) and fluorescein isothiocyanate (FITC) channels. For analysis of uptake of morpholinos by *C. parvum* sporozoites, approximately 2.5 × 10^5^ freshly purified sporozoites were suspended (in triplicate) in 300 µl of supplemented RPMI-1640 medium in 1.5 µl microfuge tubes containing 10 µM (final concentration) of fluorescein-tagged off-target standard control morpholino and 1.8 µl of Endo-Porter solution. The mixture was incubated at room temperature for 30 min and washed four times with 1000 µl of PBS by centrifugation. The final sporozoite pellet was resuspended in 20 µl of PBS and 10 µl applied onto a glass slide, covered with a coverslip and analysed by fluorescent microscope using DIC and FITC channels.

### 2.10 CpLDH and CpAMT knockdown assays

HCT-8 cells were cultured in supplemented RPMI-1640 medium in 12-well plates. When the cells were confluent, old medium was replaced with 1 ml of fresh medium containing CpLDH- or CpAMT-target vivo morpholino or off-target standard control vivo morpholino at a final concentration of 10 µM. To each well, 6 µl of Endo-Porter solution was added, and after mixing the cells were incubated at 37 °C with 5% CO_2_ for 30 min to allow uptake of vivo morpholino. Then, 1 × 10^6^ fresh sporozoites that had been incubated for 30 min in 150 µl of supplemented RPMI medium containing 10 µM of the respective vivo morpholino were added to the treated HCT-8 cultures. The mixtures were incubated at 37 °C with 5% CO_2_ for 12 h, 36 h or 56 h, then detached from the wells using a cell scraper and transferred to 15 ml conical tubes. The cells were washed three times in 5 ml of PBS and the pellet lysed in Laemmli sample buffer followed by boiling for 5 min. The samples were used for western blotting analysis to determine the knockdown effect of vivo morpholino on the targeted genes.

To determine the knockdown effect of the targeted CpLDH and CpAMT on the in vitro growth of *C. parvum*, HCT-8 cells were cultured in supplemented RPMI-1640 medium in 96-well plates. When confluent, old medium was replaced with 200 µl of fresh medium containing CpLDH- or CpAMT-target vivo morpholino or off-target standard control vivo morpholino at a final concentration of 10 µM, together with 1.2 µl of Endo-Porter solution. The cells were incubated at 37 °C with 5% CO_2_ for 30 min to allow uptake of vivo morpholino. Then, 25 µl of RPMI medium containing 5 × 10^5^ fresh sporozoites that had been incubated for 30 min with 10 µM of the respective vivo morpholino were added to the treated HCT-8 cells in triplicate. The mixtures were incubated at 37 °C with 5% CO_2_. The cells were processed for immunofluorescence assays at 36 h and 56 h p.i. following the method described by Kuhlenschmidt et al. (2016). Briefly, the medium was removed and cells were fixed with methanol-acetic acid (9:1) for 2 min at room temperature. The cells were rehydrated and permeabilised by two successive washes with wash buffer (0.1% Triton X-100, 0.35 M NaCl, 0.13 M Tris-base, pH 7.6) and blocked with 5% normal goat serum, followed by staining with antibody to *C. parvum* (SporoGlo; Waterborn, Inc. USA) overnight at 4 °C. The stained cells were washed twice with PBS followed by water, and then observed with an inverted fluorescence microscope with a 40× objective. Fluorescence quantification was done using ImageJ version 1.37v software (NIH, USA).

### 2.11 Western blot analyses

To determine the expression levels of CpLDH and CpAMT, cell lysate samples prepared as described in Section 2.9 were fractionated on SDS–PAGE and transferred onto nitrocellulose membranes. Immunoblotting was done using the monospecific purified rat anti-CpLDH or CpAMT at a dilution of 1:200 as primary antibodies, and horse radish peroxidase-conjugated chicken anti-rat (ThermoFisher Scientific, USA) as a secondary antibody at 1:2000 dilution. To check for loading, immunoblotting was done using mouse anti-*Cryptosporidium* CP15/60 sporozoite 60 K protein monoclonal antibody (LifeSpan Biosciences, Inc., USA) at 1:200 dilution as a primary antibody and horseradish peroxidase (HRP) goat anti-mouse (ThermoFisher Scientific) as a secondary antibody at 1:2000 dilution. Signal generation was performed using Clarity Western ECL Substrate (Bio-Rad) and imaging done using the FluoroChem R imager (Protein Simple, USA).

### 2.12 Statistical analyses

Statistical analyses were performed using two-tailed Student's *t* tests. *P* values of 0.05 or less were considered significant.

## 3 Results

### 3.1 Analysis of morpholino cellular toxicity and uptake

**Fig. 1:**
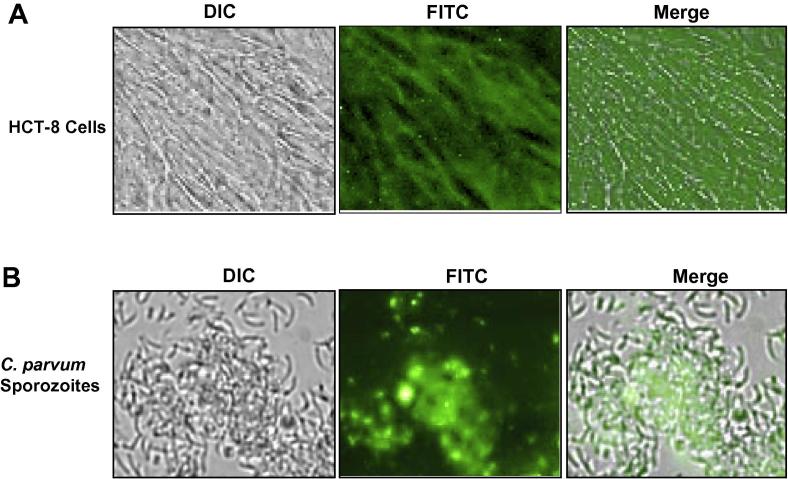
Analysis of in vitro uptake of fluorescein-tagged morpholino by HCT-8 cells and *Cryptosporidium parvum* sporozoites. (A) Confluent HCT-8 cells were cultured with 10 µM of fluorescein-tagged off-target standard control morpholino for 1 h and thereafter washed four times in PBS followed by microscopic analysis using differential interference contrast microscopy and fluorescein isothiocyanate channels. Intracellular diffuse distribution of fluorescein was observed in all the cells. (B) Freshly excysted *C. parvum* sporozoites were incubated at room temperature with 10 µM of off-target standard control morpholino for 30 min and thereafter washed four times in PBS followed by microscopic analysis using differential interference contrast and fluorescein isothiocyanate channels. Intracellular diffuse distribution of fluorescein was observed in all the sporozoites.

Prior to performing gene knockdown assays using vivo morpholinos, we derived the toxicity IC_50_ values for the CpLDH, CpAMT and standard off-target vivo morpholino in HCT-8 cells. We found that the standard off-target control, CpLDH and CpAMT vivo morpholino had IC_50_ values in HCT-8 cells of 32 ± 5.3 μM, 28 ± 3.8 μM and 29 ± 4.6 μM, respectively. Based on our previous studies involving the use of vivo morpholinos for gene knockdown in *Toxoplasma* (a protozoa that is closely related to *Cryptosporidium*), a concentration of 10 μM of vivo morpholino was found to be optimum for effective gene knockdown in vitro (Lai et al., 2012, Witola et al., 2014). Thus, we chose to start with this concentration to determine the uptake of morpholinos by HCT-8 cells and *C. parvum* sporozoites. However, for uptake studies, we used morpholino bearing a fluorescein tag at the 3′-terminus (to facilitate visualisation of morpholino), but without the octa-guanidine dendrimer delivery moiety tag (which facilitates cellular uptake) since morpholinos bearing both tags are unavailable (Gene Tools Inc.). Thus, to facilitate cellular uptake of fluorescein-tagged morpholinos, we added Endo-Porter solution to the assay as recommended by the manufacturer (Gene Tools Inc.). We observed that within 1 h of incubation with morpholino, HCT-8 cells internalised substantial amounts of morpholino, with the morpholino being diffusely distributed throughout the cytosol of the cells (Fig.1A). Uptake of morpholino by HCT-8 cells after incubation periods longer than 1 h was not significantly higher than that observed at 1 h (data not shown). Further, we found that *C. parvum* sporozoites internalised substantial amounts of morpholino at a concentration of 10 μM within 30 min, with the morpholino distributing diffusely within the parasite cytosol (Fig.1B). Incubation for periods longer than 30 min did not result in significantly higher amounts of internalised morpholino than for 30 min (data not shown).

### 3.2 CpLDH and CpAMT cloning, expression, purification and production of antisera

**Fig. 2:**
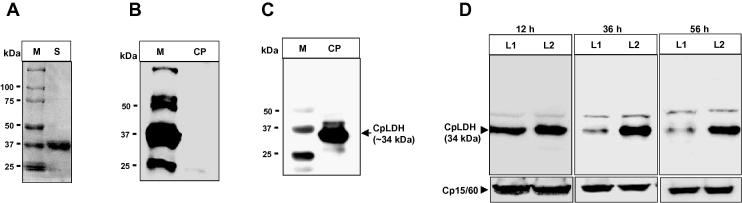
Western blot analyses of *Cryptosporidium parvum* lactate dehydrogenase antisera specificity and knockdown effect of *C. parvum* lactate dehydrogenase-target vivo morpholino. (A) SDS–PAGE analysis of column affinity-purified recombinant *C. parvum* lactate dehydrogenase protein (stained with Coomassie blue) that was used to generate rat anti-*C. parvum* lactate dehydrogenase antibodies. Lane M, protein ladder; lane S, CpLDH protein. (B) Western blotting analysis of *C. parvum* lysate using rat pre-immune antisera, and (C) using rat anti-sera generated against recombinant *C. parvum* lactate dehydrogenase protein. Lane M, protein ladder with 50, 37 and 25 kDa marker bands shown; lane CP, *C. parvum* protein lysate with the recognised *C. parvum* lactate dehydrogenase protein band shown at approximately 34 kDa. (D) Analysis of the knockdown effect of *C. parvum* lactate dehydrogenase-target vivo morpholino on the expression of *C. parvum* lactate dehydrogenase protein in *C. parvum* sporozoites cultured for 12 h, 36 h and 56 h in HCT-8 cells. Lane L1, protein lysate of infected HCT-8 cells treated with *C. parvum* lactate dehydrogenase-target vivo morpholino; lane L2, protein lysate of infected HCT-8 cells treated with off-target standard control vivo morpholino. As a loading control, Cp15/60 shows equal amounts of the protein lysates blotted using antibody against *C. parvum* Cp15/60 sporozoite protein at the different time points.

**Fig. 3:**
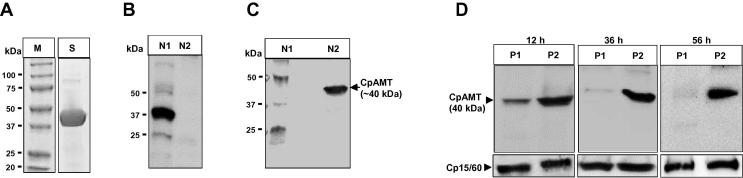
Western blot analyses of *Cryptosporidium parvum* putative arginine n-methyltransferase antisera specificity and knockdown effect of *C. parvum* putative arginine n-methyltransferase-target vivo morpholino. (A) SDS–PAGE analysis of column affinity-purified recombinant *C. parvum* putative arginine n-methyltransferase protein (stained with Coomassie blue) that was used to generate rat anti-*C. parvum* putative arginine n-methyltransferase antibodies. Lane M, protein ladder; lane S, CpAMT protein. (B) Western blotting analysis of *C. parvum* lysate using rat pre-immune antisera, and (C) using rat anti-sera generated against *C. parvum* putative arginine n-methyltransferase protein. Lane N1, protein ladder with 50, 37 and 25 kDa marker bands shown; lane N2, *C. parvum* protein lysate with the recognised *C. parvum* putative arginine n-methyltransferase protein band shown at approximately 40 kDa. (D) Analysis of the knockdown effect of *C. parvum* putative arginine n-methyltransferase-target vivo morpholino on the expression of *C. parvum* putative arginine n-methyltransferase protein in *C. parvum* sporozoites cultured for 12 h, 36 h and 56 h in HCT-8 cells. Lane P1, protein lysate of infected HCT-8 cells treated with *C. parvum* putative arginine n-methyltransferase-target vivo morpholino; lane P2, protein lysate of infected HCT-8 cells treated with off-target standard control vivo morpholino. As a loading control, Cp15/60 shows equal amounts of the protein lysates blotted using antibody against *C. parvum* Cp15/60 sporozoite protein at the different time points.

The coding sequence of CpLDH cloned from *C. parvum* (AUCP-1 isolate) was 966 bp long and 99.79% identical to that reported in GenBank (accession number AF274310.1). It coded for a 321 amino acids long protein that, when compared with the protein sequence of CpLDH reported in GenBank, had amino acid residue substitutions of F-198-L, R-251-K and K-295-E. The recombinant CpLDH protein that was expressed and purified from *E. coli* was approximately 34 kDa, consistent with the expected molecular size (Fig.2A). The cloned CpAMT sequence was 1047 bp long, coding for a 348 amino acid protein sequence that was 100% identical to that reported in Cryptodb (Cgd8_4760). The recombinant CpAMT expressed and purified from *E. coli* was approximately 40 kDa, consistent with the expected molecular weight (Fig.3A). The CpLDH and CpAMT recombinant proteins were used to generate polyclonal antibodies in rats that were subsequently purified to monospecificity. Rat pre-immune sera did not recognise recombinant CpLDH protein (Fig.2B) nor CpAMT protein (Fig.3B). The monospecific antibodies recognised the respective recombinant proteins on western blotting, and importantly, recognised their respective endogenous proteins in *C. parvum* oocysts lysate that were consistent with the expected molecular sizes of 34 and 40 kDa for CpLDH and CpAMT, respectively (Figs. 2C, 3C).

### 3.3 CpLDH and CpAMT morpholinos down-regulate expression of target protein in *C. parvum*

To determine the effect of CpLDH- and CpAMT-target vivo morpholinos on the expression of their respective target proteins in *C. parvum* cultured in HCT-8 cells, cultures treated with target vivo morpholino or standard off-target control vivo morpholino for 12 h, 36 h or 56 h were harvested and equal amounts of whole cell lysates analysed by western blotting. Prior to infection of HCT-8 cells with *C. parvum* sporozoites, the parasites and cells were separately treated with 10 μM vivo morpholino for 30 min to facilitate advance internalisation of vivo morpholino. The vivo morpholino treatment was maintained in the infected cultures and the sporozoites were allowed to establish infection in HCT-8 cells before harvesting at the respective time points of 12 h, 36 h or 56 h p.i. We found that CpLDH- and CpAMT-target vivo morpholinos significantly reduced the expression of their target proteins in the parasites after 36 h and 56 h of culture in a time-dependent manner compared with the off-target vivo morpholino (Figs. 2D, 3D). By densitometric analysis of the western blot protein bands, the CpLDH-target morpholino reduced the CpLDH protein amount by eight-fold (±1.0), while the CpAMT-target vivo morpholino reduced the amount of CpAMT protein by nine-fold (±0.5). A faint protein band (above the 34 CpLDH protein band) was detected in the *C. parvum* lysates blotted with anti-CpLDH antibodies (Fig.2D). The size of this faint band was not consistent with the expected molecular size of CpLDH, and its expression was not affected by CpLDH-target vivo morpholino treatment, indicating that it was non-specific. When western blotting was done on equivalent amounts of the protein lysates using antibody against *C. parvum* Cp15/60 sporozoite protein that was not targeted by the vivo morpholinos, there was no notable difference in the protein levels (Figs. 2D, 3D), indicating that the CpLDH and CpAMT vivo morpholinos were specific in their knockdown effect. By bioinformatics analysis, both CpLDH and CpAMT do not have significant homologies in the human (from which host HCT-8 cells were derived) genome that would facilitate specific binding of the CpLDH or CpAMT target morpholinos to any transcripts in the human transcriptome. Treatment of the parasite cultures with concentrations of vivo morpholino above 10 μM did not significantly decrease the target proteins below those observed at 10 μM of vivo morpholino (data not shown), and therefore we decided to use 10 μM as the concentration of choice in subsequent experiments.

### 3.4 CpLDH knockdown decreases *C. parvumm* growth in HCT-8 cells

**Fig. 4:**
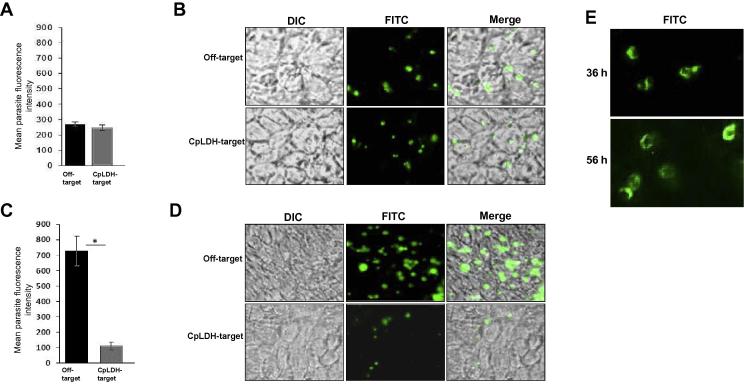
Analysis of the effect of *Cryptosporidium parvum* lactate dehydrogenase knockdown on the growth of *C. parvum* in HCT-8 cells as determined by immunofluorescence assays*.* (A) Quantification of *C. parvum* growth in cultures treated with control off-target vivo morpholino (Off-target) or with *C. parvum* lactate dehydrogenase-target vivo morpholino (CpLDH-target) for 36 h. The fluorescence generated by the parasites is proportional to the amount of parasites in culture and is depicted as the mean relative fluorescence intensity on the *Y*-axis. (B) Representative images of immunofluorescence staining of the *C. parvum*-infected HCT-8 cells incubated with vivo morpholinos for 36 h. (C) Quantification of *C. parvum* growth in cultures treated with control off-target vivo morpholino or with *C. parvum* lactate dehydrogenase-target morpholino for 56 h. (D) Representative images of immunofluorescence staining of the *C. parvum*-infected HCT-8 cells incubated with vivo morpholino for 56 h. Green fluorescence depicts intracellular *C. parvum* merozoites (×40 objective). (E) Representative high magnification (×63 objective) fluorescein isothiocyanate images showing the morphology of the intracellular *C. parvum* merozoites at 36 h and 56 h p.i. The data shown represent means of three independent experiments with S.E. bars and levels of statistical significance between groups indicated by an asterisk (*, *P* < 0.05).

Having determined the non-toxic concentration of vivo morpholinos that could significantly knockdown the expression of CpLDH and CpAMT during in vitro culture of *C. parvum* in HCT-8 cells, we investigated the effect of CpLDH and CpAMT knockdown on the survival and growth of *C. parvum* in culture. Even though treatment of infected HCT-8 cells with CpLDH vivo morpholino resulted in notable knockdown of CpLDH expression, there was no significant difference in the growth of parasites between the control vivo morpholino-treated and the CpLDH vivo morpholino-treated parasites at 36 h post-infection (Fig.4A, B). This was consistent with the western blotting results that showed a reduction in CpLDH signal but without a corresponding decrease in the non-targeted Cp15/60 sporozoite protein which was used as the parasite protein loading control. However, at 56 h p.i., there was a seven-fold reduction in parasite growth (Fig.4C, D), indicating that CpLDH knockdown affected parasite growth at an advanced stage of culture. Analysis of the effect of CpAMT knockdown did not show any effect on parasite growth, both at 36 h and 56 h of culture (data not shown) even though a significant reduction was found in CpAMT protein expression as early as 36 h of culture.

## 4 Discussion

With the successful completion and annotation of the *Cryptosporidium* genome, it has become clear that this protozoa lacks conventional drug targets that are currently being pursued as molecular drug targets in other protozoan parasites such as *Plasmodium* and *Toxoplasma* (Abrahamsen et al., 2004). Nevertheless, the complete genome sequence of *Cryptosporidium* has unveiled numerous potential molecular targets that include several plant-like and bacteria-like enzymes (Abrahamsen et al., 2004). These seemingly potential molecular drug targets in *Cryptosporidium* will need to be functionally characterised using genetic tools in order to be validated. However, at present there are extremely limited genetic tools for functional analysis and validation of possible molecular targets in *Cryptosporidium* (Vinayak et al., 2015). This is further complicated by the lack of a continuous in vitro culture system for *Cryptosporidium*. Nevertheless, there have been recent reports on the development of engineered cell culture systems for in vitro continuous culture of *C. parvum* (Morada et al., 2016, DeCicco RePass et al., 2017).

Recent advances in use of vivo morpholinos for gene silencing in protozoa (Lai et al., 2012, Witola et al., 2014, Garg et al., 2015) provide the potential of using this approach for gene manipulation in *Cryptosporidium*. Vivo morpholinos are designed and chemically synthesised for targeted gene silencing without the need for prior processing by cellular enzymes. They are designed to be stable and to readily cross multiple cellular membranes without the need of electroporation or use of other transfection procedures that are deleterious to cells (Moulton et al., 2004; Li and Marcos, 2008). Because vivo morpholinos are relatively non-toxic and non-immunogenic, they can allow in vivo studies using murine infection models. Although there is no tissue culture system for continuous passage, *C. parvum* can be cultured and undergo development in vitro for approximately 3 days by infecting human HCT-8 cells. The 3 days of culture provides a window for effective gene knockdown using vivo morpholinos in *C. parvum*.

Herein, we have developed an assay using vivo morpholinos for targeted significant inhibition of protein translation within the first 36 h of in vitro culture of *C. parvum* in HCT-8 cells. Our data show that morpholinos at non-toxic concentrations are rapidly internalised by both *C. parvum* and host cells, and distribute diffusely throughout the cytoplasm of the parasites and cells. Diffuse distribution of morpholinos throughout cell cytosol is considered an indication of successful and effective delivery into the target cell (Morcos, 2001). The uptake of morpholinos by cells is by endocytosis, resulting in the compartmentalization of morpholinos in intracellular endosomes shortly after uptake. For the morpholinos to be effective, they have to be released from the endosomes and distribute diffusely throughout the cell cytosol (Morcos, 2001). Considering the limited period (approximately 3 days) that *C. parvum* can be maintained in culture, to maximise the effect of morpholinos, we endeavoured to deliver those into the parasites and host cells separately before performing the infection assay. We achieved effective targeted gene knockdown for both CpLDH and CpAMT during the first half (36 h) of the culture period, which still left enough time for continued observation of the effect of the knockdown on parasite growth within the 3 day period in which *C. parvum* can be grown in culture. Unlike the unstable small interfering RNAs that are used for gene knockdown by RNA interference, morpholinos are very stable and not easily degradable because, structurally, they contain deoxyribonucleic instead of ribonucleic bases. Additionally, the phosphorodiamidate groups on morpholinos are neutral and hydrophilic, making them highly stable and water-soluble (Moulton et al., 2004). For this reason, they are likely to persist in culture, thereby maintaining a sustained knockdown effect without replenishment.

Of the two genes that we targeted, we found that knockdown of CpLDH produced a notable dramatic reduction in parasite growth at 56 h of culture. When observed at an earlier stage (36 h) of culture, however, there was no notable difference in the intensities of parasites in CpLDH-knockdown and control cultures, despite significant down-regulation of the CpLDH protein. The CpLDH protein in *C. parvum* is a bacteria-type lactate dehydrogenase enzyme that the parasite uses to generate metabolic energy (ATP) in the glycolytic pathway (Madern et al., 2004, Zhang et al., 2015), since *C. parvum* lacks both the Krebs cycle and the cytochrome-based respiration chain (Abrahamsen et al., 2004). In extracellular sporozoites and merozoites, CpLDH has been shown to be localised in the cytosol (Zhang et al., 2015). However, once *C. parvum* infects host cells and forms the parasitophorous vacuole membrane (PVM), CpLDH becomes associated with the PVM, implying that the parasite PVM is involved in the generation of ATP (Zhang et al., 2015). During the first 36 h after infection of the host cells, the parasites would have completed the first generation of merogony but still be relatively few in number. However, as they progress into the second generation of merogony, the number and size of merozoites increases, extending the size of the PVM, and this in turn would require more CpLDH to generate the much needed metabolic energy to fuel parasite growth. This is consistent with our observation that at 56 h of culture (corresponding to the later stage of the second generation of merogony), there were significantly fewer and smaller parasites in CpLDH-knockdown than in the control cultures. Corroborating our observations, Zhang et al. (2015) have shown that culturing *C. parvum* in the presence of a CpLDH inhibitor (gossypol) decreased the growth of the parasites in a time-dependent manner, with a more pronounced reduction being observed in the later stages of culture. Thus, our findings indicate that CpLDH plays an essential role in fueling the intracellular growth and development of *C. parvum*.

By targeting CpAMT using vivo morpholino, we also successfully knocked down the expression of the CpAMT protein within the first 36 h of culture. However, there was no notable effect of CpAMT knockdown on the growth and development of *C. parvum* in culture throughout the culture period of 3 days. Nevertheless, effective knockdown of both CpLDH and CpAMT provides the proof-of-principle that the vivo morpholino knockdown assay in *C. parvum* is consistent. Together, our findings provide a gene regulation approach for interrogation of gene function in *Cryptosporidium* in vitro. Further, this study has provided genetic evidence for the essential role of CpLDH in the growth and development of *C. parvum*.
